# Design and study protocol for a cluster randomized trial of a multi-faceted implementation strategy to increase the uptake of the USPSTF hypertension screening recommendations: the EMBRACE study

**DOI:** 10.1186/s13012-020-01017-8

**Published:** 2020-08-08

**Authors:** Nathalie Moise, Erica Phillips, Eileen Carter, Carmela Alcantara, Jacob Julian, Anusorn Thanataveerat, Joseph E. Schwartz, Siqin Ye, Andrea Duran, Daichi Shimbo, Ian M. Kronish

**Affiliations:** 1 Center for Behavioral Cardiovascular Health, Department of Medicine, Columbia University Irving Medical Center, 622 W. 168th Street, New York, NY 10032 USA; 2 Division of General Internal Medicine, Weill Cornell Medicine, 1320 York Avenue, New York, NY 10021 USA; 3 Columbia University School of Nursing, Columbia University Irving Medical Center, 560 West 168th Street, New York, NY 10032 USA; 4 Columbia University School of Social Work, 1255 Amsterdam Avenue, New York, NY 10027 USA; 5 Department of Psychiatry and Behavioral Science, Stony Brook University School of Medicine, 101 Nicolls Road, Stony Brook, NY 11794 USA

**Keywords:** Hypertension, Out-of-office testing, Primary health care

## Abstract

**Background:**

The US Preventive Services Task Force (USPSTF) recommends out-of-office blood pressure (BP) testing to exclude white coat hypertension prior to hypertension diagnosis. Despite improved availability and coverage of home and 24-h ambulatory BP monitoring (HBPM, ABPM), both are infrequently used to confirm diagnoses. We used the Behavior Change Wheel (BCW) framework, a multi-step process for mapping barriers to theory-informed behavior change techniques, to develop a multi-component implementation strategy for increasing out-of-office BP testing for hypertension diagnosis. Informed by geographically diverse provider focus groups (*n* = 63) exploring barriers to out-of-office testing and key informant interviews (*n* = 12), a multi-disciplinary team (medicine, psychology, nursing) used rigorous mixed methods to develop, refine, locally adapt, and finalize intervention components.

The purpose of this report is to describe the protocol of the Effects of a Multi-faceted intervention on Blood pRessure Actions in the primary Care Environment (EMBRACE) trial, a cluster randomized control trial evaluating whether a theory-informed multi-component strategy increased out-of-office testing for hypertension diagnosis.

**Methods/design:**

The EMBRACE Trial patient sample will include all adults ≥ 18 years of age with a newly elevated office BP (≥ 140/90 mmHg) at a scheduled visit with a primary care provider from a study clinic. All providers with scheduled visits with adult primary care patients at enrolled ACN primary care clinics were included. We determined that the most feasible, effective implementation strategy would include delivering education about out-of-office testing, demonstration/instruction on how to perform out-of-office HBPM and ABPM testing, feedback on completion rates of out-of-office testing, environmental prompts/cues via computerized clinical decision support (CDS) tool, and a culturally tailored, locally accessible ABPM testing service. We are currently comparing the effect of this locally adapted multi-component strategy with usual care on the change in the proportion of eligible patients who complete out-of-office BP testing in a 1:1 cluster randomized trial across 8 socioeconomically diverse clinics.

**Conclusions:**

The EMBRACE trial is the first trial to test an implementation strategy for improving out-of-office testing for hypertension diagnosis. It will elucidate the degree to which targeting provider behavior via education, reminders, and decision support in addition to providing an ABPM testing service will improve referral to and completion of ABPM and HBPMs.

**Trial registration:**

Clinicaltrials.gov, NCT03480217, Registered on 29 March 2018

Contributions to the literature

- Few if any prior studies have identified an effective and sustainable model for implementing out-of-office testing for hypertension diagnosis, which has become essential in the era of telemedicine.
- Implementation scientists have called for rigorous approaches to operationalizing the selection of implementation strategies. We describe a rigorous, multi-disciplinary approach to employing the Behavior Change Wheel for intervention mapping.
- We demonstrate that in theory a multi-component implementation strategy of provider education, reminders, and decision support coupled with an ABPM testing service would be needed to implement out-of-office BP testing for the diagnosis of hypertension.

## Introduction

Hypertension is one of the most common reasons for primary care visits and a leading risk factor for cardiovascular disease and all-cause mortality in the USA [1–5]. However, there are challenges to screening for hypertension and measuring blood pressure (BP) in the office setting that make incorrect or overdiagnosis common, leading to unnecessary treatment with BP medications and wasteful healthcare utilization [6–8]. A systematic review conducted by the US Preventive Services Task Force (USPSTF) found that 5–65% of patients with elevated office BP do not have high BP when measured out of the office [9]—a BP phenotype known as *white coat hypertension* [10]. In comparison with sustained hypertension (elevated BP in and out of the office settings), the preponderance of evidence has suggested that white coat hypertension does not confer substantial cardiovascular risk and treatment does not improve prognosis [11–13].

Based on these data, the USPSTF, a key source for evidence-based recommendations for clinical preventive services in the USA, updated their hypertension screening recommendations in 2015 to advise out-of-office BP measurements for diagnostic confirmation of hypertension before initiating treatment (grade A recommendation; high certainty of a substantial net benefit). This recommendation is consistent with those of other US-based (e.g., American College of Cardiology/American Heart Association (ACC/AHA), American Society of Hypertension) and international societies (e.g., U.K.’s National Institute of Clinical Excellence, European Society of Hypertension/European Society of Cardiology) [14–17].

Out-of-office BP testing can be performed using home BP monitoring (HBPM) [18] or ambulatory BP monitoring (ABPM) [19]. HBPM involves patients self-measuring their BP using an automated home BP device while seated and resting, twice per day across 1 week and then averaging the BP readings obtained. In contrast, ABPM involves having a BP cuff placed on a patient’s upper arm for 24 h, with the cuff automatically inflated at regular intervals (e.g., every 15–30 min) to give multiple BP readings in the context of a patient’s everyday life, including during sleep [9, 11]. Despite the availability of affordable HBPM devices and the availability of reimbursement for ABPM by many health insurers, both are infrequently utilized to confirm diagnoses of hypertension in the USA; for example, just 0.1% of Medicare beneficiaries had a claim submitted for ABPM from 2007 to 2010) [20–23]. Thus, adherence to the updated hypertension screening guidelines requires a major paradigm shift in how primary care providers diagnose hypertension in the USA (i.e., no longer relying solely on office BP) [24].

In spite of this marked underuse of out-of-office BP testing and increasing need in the post-COVID era of telemedicine/remote care, there have been few if any studies of interventions to increase the use of guideline-recommended out-of-office BP testing, either ABPM or HBPM, to establish the diagnosis of hypertension. Furthermore, to our knowledge, an implementation science approach to developing an effective and sustainable model for implementing out-of-office testing has not been applied, providing an opportunity to not only elucidate how best to operationalize the selection of multi-component implementation strategies, a key gap in the implementation science literature [25, 26], but also how best to rigorously design BP trials in real world settings, a key gap in the hypertension literature. We therefore used a multi-disciplinary stakeholder approach to employing the Behavior Change Wheel (BCW) framework [27] for the purposes of developing a theory-informed implementation strategy that would address perceived provider barriers to increasing the uptake of out-of-office BP testing as part of hypertension screening and diagnosis. Our objective is to test its effectiveness through the Effects of a Multi-faceted intervention on Blood pRessure Actions in the primary Care Environment (EMBRACE) trial, a cluster randomized trial across a network of 8 primary care clinics. The implementation strategy as well as protocol and analysis plan for the EMBRACE trial are described here. Our results will inform efforts to increase out-of-office BP testing for the diagnosis of hypertension and will have wide ranging implications pertinent to the implementation of current hypertension screening guidelines [14–16].

## Methods

### Overview

We endeavored to develop a theory-informed, scalable implementation strategy to address barriers to increasing the uptake of the recent USPSTF hypertension recommendation, which represents a paradigm shift in the diagnosis of hypertension (i.e., move from relying on office BP to out-of-office testing). To develop a theory-informed strategy, we drew primarily on Michie and colleagues’ BCW framework [27]. We chose this framework because it links identified behavioral targets to intervention functions most likely to bring about clinic and provider level change, and it has increasingly been used to develop implementation strategies [28]. We employed a multi-disciplinary stakeholder process to operationalize this multi-step process and developed a multi-component implementation strategy for increasing the completion of both ABPM and HBPM testing for the purposes of hypertension diagnosis. We are now conducting a cluster randomized trial (CRT) testing using this multi-faceted strategy aimed at increasing implementation of the USPSTF hypertension screening guideline.

### Implementation strategy design

#### Understand target behavior

The BCW framework first prompts one to identify both a primary behavior and the barriers related to the capability, opportunity, and/or motivation needed to influence that target behavior (COM-B) (Additional file 1) [27]. To identify behavioral targets, we conducted qualitative assessments of patient and primary care provider attitudes toward completing out-of-office BP testing. Our now published analyses of patient focus groups (3 focus groups involving 20 Spanish- and English-speaking patients) revealed widespread comprehension of the concept white coat hypertension and acceptability of out-of-office BP testing, with patients overwhelmingly believing that they could successfully complete both forms of out-of-office BP testing if recommended by their provider [29]. In contrast, nominal groups (i.e., structured variation of a small-group discussion that elicits and ranks individual attitudes followed by consensus reaching around key themes) [30, 31] with primary care providers (9 groups involving 63 providers in geographically diverse settings) revealed major provider-level barriers to successful ordering of ABPM and HBPM for hypertension diagnosis [32]. Thus, our qualitative studies revealed that provider behavior (e.g., test ordering) should be the key behavioral target to drive the evidence implementation gap around the completion of out-of-office BP testing for hypertension diagnosis [27].

Consistent with the BCW framework, barriers elicited in our nominal groups with primary care providers were coded by members of the research team into COM-B categories (Table 1) [32]. Differences in coding were reconciled through discussion and consensus, with researchers referring to original transcripts to reassess the context of the codes. Top-ranked provider barriers to completing out-of-office BP testing included lack of knowledge about the guideline (capability); limited time to instruct patients about HBPM, costs of home BP devices, and challenges with accessing ABPM testing (opportunity); and concerns about their ability to get patients to successfully and accurately complete tests and about whether the benefits of testing outweighed the costs (motivation).

**Table 1:** Primary care providers’ perceptions of key barriers to completing ambulatory blood pressure monitoring and home blood pressure monitoring^a^

	ABPM barriers	HBPM barriers
Psychological and physical capability	• Do not know how to order the test • Do not know how to place ABPM device on patients • Do not know how to interpret ABPM results • Insufficient training in how to explain results to patients • Lack of awareness of guidelines • Lack of knowledge about the indications for testing	• Do not know how to train patients to conduct HBPM testing • Do not know how to review and interpret HBPM results • Do not know the protocol for HBPM testing
Physical and social opportunity	• Complicated process to get insurance coverage • Out-of-pocket costs • Complex logistics of ordering the test • Limited access to ABPM testing • Cost of ABPM equipment • Lack of staff time to handle the process • Lack of physician time to communicate the need and process to patients • Lack of physician time to manage and interpret the data	• Out-of-pocket cost of HBPM device • Low reimbursement to physicians • Lack of time to train patients in HBPM protocol • Lack of time to review HBPM results • Lack of time to follow-up on technical and clinical problems arising during measurement
Reflective and automatic motivation	Provider perceptions that: • Patients will be unwilling to perform the test • Patients will be unable to complete the test due to discomfort and lack of time to return the device • Test results will not be accurate due to patient non-compliance with testing protocol • Test results will not be accurate due to inconsistencies in how data are cleaned and interpreted • Testing is not cost-effective • Test results will not be sufficient to exclude white-coat hypertension • Test will not improve patient outcomes • Test will lead to unnecessary delays in hypertension treatment	Provider perceptions that: • Patients will be unable to complete HBPM testing due to low health literacy, time requirement, intrusiveness of testing, requirement of a routine, or requirement to bring HBPM results to the office • Test results will not be accurate due to use of invalid HBPM devices • Test results will not be accurate due to patient non-compliance with HBPM protocol (e.g., wrong cuff size, wrong timing of blood pressure readings) • Test results will not be accurate due to patient factors such as body habitus • Testing could increase patient anxiety and hence, accuracy of test results

*ABPM* ambulatory blood pressure monitoring, *HBPM* home blood pressure monitoring

^a^Adapted from results presented in *Kronish et al. (2017), J Am Soc Hypertens* (categorization into *Capability, Opportunity, Motivation* (COMB) constructs is novel for this study)

#### Identify intervention options, components, and mode of delivery

The research team next used the BCW to facilitate the mapping of COM-B barriers to corresponding intervention functions (i.e., 9 broad categories by which an intervention can change behavior, e.g., education, training, persuasion), policy categories (i.e., 7 policies representing types of decisions made by authorities that help to support and enact interventions), and behavior change techniques (BCTs; i.e., a standardized language for describing the active ingredients in behavior change interventions via which intervention functions and policy categories are delivered; Additional file 1). Given that several interventions, policies, and techniques mapped to each COM-B construct, the BCW prompts intervention developers to select only those intervention components deemed affordable (within an acceptable budget for all), practical (can be delivered as designed), efficacious (effective and cost-effective related to designed objectives in real world context), acceptable (judged appropriate by relevant stakeholders), safe (no unwanted side effects), and equitable (does not increase disparities) [APEASE criteria] [27]. After meeting to calibrate definitions and ensure reliable understanding of APEASE criteria, four members of the research team (N.M., I.K., C.A., E.S.) from multiple disciplines (medicine, psychology, nursing) independently reviewed the feasibility of mapped intervention functions, policy categories, and behavior change techniques. For example, three intervention functions (coercion, restriction, and incentivization) and three policy categories (fiscal, regulation, legislation) were viewed as not meeting APEASE criteria (Table 2).

**Table 2:**
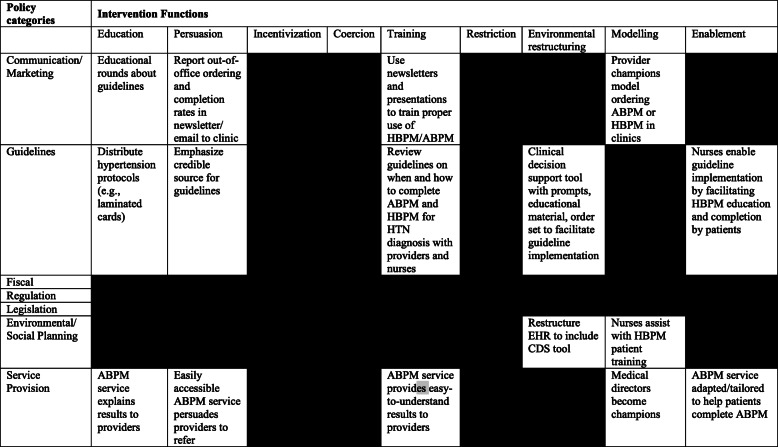
Mapping intervention functions to policy categories to identify feasible and relevant intervention components

Black cells indicate that intervention functions and policy categories were not deemed feasible and relevant by one or more APEASE criteria

*APEASE* acceptability, practicability, effectiveness/cost-effectiveness, affordability, safety/side-effects, equity; *ABPM* ambulatory blood pressure monitoring; *HBPM* home blood pressure monitoring; *HTN* hypertension, *EHR* electronic health record; *CDS* clinical decision support

*Black* = guidelines and intervention functions do not map or map but do not meet APEASE criteria

*White* = map and deemed feasible and relevant by APEASE criteria

Through iterative group discussions and continuous review of primary qualitative data collected, we determined that the most feasible and effective behavioral change techniques would include (1) delivering information about the benefits and costs of out-of-office testing, (2) demonstration/instruction on how to order out-of-office HBPM and ABPM testing, (3) feedback on completion rates and outcomes of out-of-office BP testing, and (4) adding objects to the environment including an electronic computerized clinical decision support (CDS) tool that would provide prompts and cues to follow the new guideline. Within policy considerations, service provision was considered a key delivery mechanism for several intervention functions. Thus, adapting and tailoring a pre-existing Columbia University ABPM testing service, called ActiveBP, was selected as another important intervention component. Key service modifications included an easy test ordering process, creation of educational materials (that address numeracy, literacy, language) for patients and providers in English and in Spanish; easy-to-understand reports that summarize test results; and a streamlined insurance look-up and billing process to reduce the logistical hurdles for completing ABPM testing. The multi-disciplinary implementation team additionally determined that a multi-pronged mode of delivery involving lectures, websites, e-mails, and the EHR would maximally reach primary care providers who were the primary target of the intervention (Table 3).

**Table 3:**
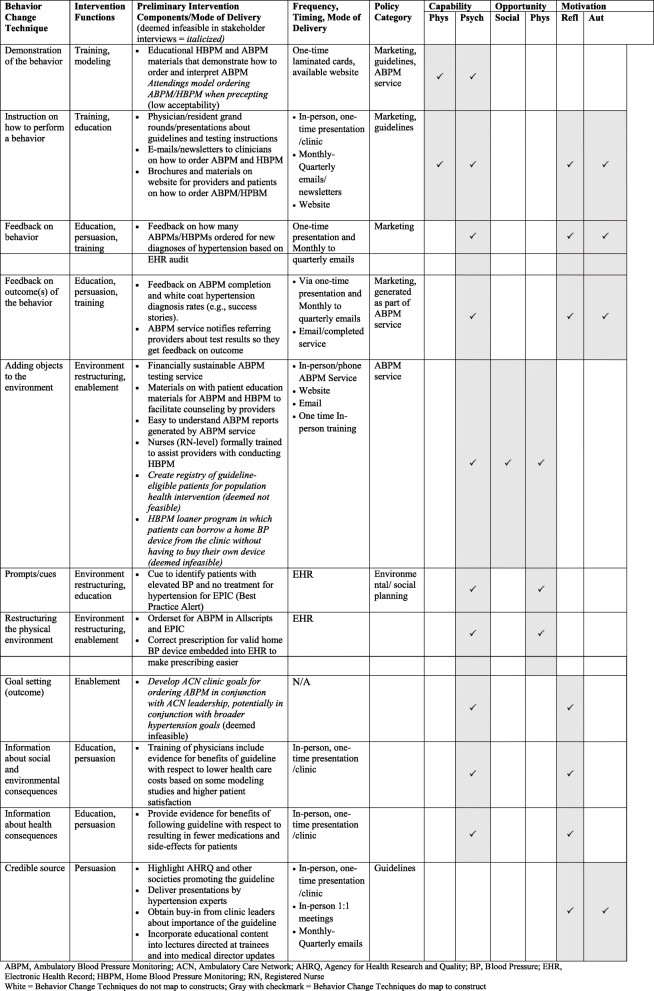
Provider/practice level intervention development process using the behavior change wheel

*ABPM* ambulatory blood pressure monitoring, *ACN* ambulatory care network, *AHRQ* Agency for Health Research and Quality, *BP* blood pressure, *EHR* electronic health record, *HBPM* home blood pressure monitoring, *RN* registered nurse

*White* = behavior change techniques do not map to constructs, *gray with checkmark* = behavior change techniques do map to construct

#### Finalizing multi-component strategy

To confirm the feasibility and acceptability of strategy components from the perspective of providers and administrators in the local clinical context, we conducted key informant interviews with health system leaders (*n* = 3), medical directors (*n* = 2), primary care attendings (*n* = 2), trainees (*n* = 2), clinic administrators (*n* = 1), medical assistants (*n* = 1), patient financial advisors (i.e., patient-facing front desk staff, *n* = 1), and registered nurses (RNs, *n* = 1). We used snowball techniques to identify interviewees and continued until we reached saturation (i.e., additional interviews ceased to identify additional themes) [33]. Key stakeholder interviews were professionally transcribed and coded by two authors (J.J. and I.K.) using content analysis, with discrepancies reconciled through consensus. There was broad consensus for most strategies and modes of delivery with the exception of CDS tools. Further, key stakeholders agreed that an ABPM testing service would be necessary as having individual providers or clinics offer ABPM testing on site would not be feasible.

Acceptability of a computerized CDS tool as a mode of delivery for education and prompts/cues varied. Two key stakeholders expressed reservations about using automated alerts, noting that these were often ignored due to alert-fatigue among providers and could even be viewed as overly intrusive. Other stakeholders, however, held more favorable views toward automated alerts if paired with education on their use. In contrast to automated alerts, there was unanimous agreement over less intrusive CDS tools, such as incorporating orders for ABPM into commonly used EHR order sets. This led to the development of a scaled-down version of a CDS tool, without intrusive alerts. Other than the CDS tools, strategy components did not substantially change after key stakeholder interviews (Table 3).

### Study design

#### Study setting

A trial evaluating this multi-component implementation strategy is now being conducted at eight primary care clinics that are part of the Ambulatory Care Network (ACN) of New York-Presbyterian Hospital (NYPH), which is affiliated with two academic medical centers–Weill Cornell Medicine and Columbia University Irving Medical Center. The ACN includes 10 adult primary care clinics distributed throughout upper Manhattan and serves a predominantly low-income, publicly insured population with a large proportion of Hispanics and African Americans. The primary care clinics are staffed by a mix of internal medicine physicians, family practitioners, geriatricians, nurse practitioners, and graduate medical education (GME) trainees. The clinics use two different electronic health records (EHRs)—Allscripts (Allscripts Sunrise, Allscripts, Chicago, IL) and EPIC (EPIC systems, Verona, IL)—two of the largest health information technology systems in the USA.

#### Ethical considerations

The trial was approved by the institutional review boards (IRB) of Columbia University Irving Medical Center and Weill Cornell Medicine. The trial was also registered at clinicaltrials.gov (NCT03480217).

#### Eligibility

Primary care clinics that served adult patients and were part of the NYPH ACN were eligible for this trial. To be included, the medical director of the clinic had to agree to participation in the trial. Two clinics that were primarily staffed by internal medicine residents—one affiliated with Weill Cornell and one with Columbia—were reserved for implementation development (e.g., focus groups, stakeholder interviews) and were excluded.

All providers who had scheduled visits with adult primary care patients at enrolled ACN primary care clinics were included. Patients were included if they were at least 18 years old and had a newly elevated office BP (systolic BP ≥ 140 mmHg or diastolic BP ≥ 90 mmHg) at a scheduled visit with a primary care provider from a study clinic (i.e., screened positive for hypertension based on office BP and eligible for out-of-office BP testing). Consistent with the USPSTF hypertension screening recommendations, patients were excluded if they had (1) prior diagnosis of hypertension; (2) prior diagnosis of white coat hypertension; (3) prior prescribed antihypertensive medication; (4) repeat manual office BP < 140/90 mmHg; (5) severely elevated office BP (systolic BP ≥ 180 mmHg or diastolic BP ≥ 110 mmHg); or (6) evidence of target-organ damage (chronic kidney disease with creatinine > 1.5 mg/dL or prior history of stroke, transient ischemic attack, coronary artery disease, myocardial infarction, congestive heart failure, or peripheral arterial disease). Determinations of patient eligibility were based on what was recorded in the EHR.

#### Enrollment, randomization, and allocation

The study principal investigator (I.K.) met with the medical directors of each of the eight potentially eligible ACN clinics to obtain verbal consent for their participation. Medical directors of all eight eligible primary care clinics agreed to adopt and be enrolled in the trial.

The 8 clinics were then matched in pairs according to clinic size and patient characteristics (i.e., the two HIV clinics were matched; the two smaller-sized community-based internal medicine clinics were matched; the two larger-sized academic clinics with substantial numbers of trainees were matched, the remaining medium-sized primary care clinics were matched). Within each pair, one clinic was randomized to the intervention and the other to the wait-list control condition. Randomization was generated using a random number generator in SAS version 9.4. Clinic assignments were made by a study statistician who had no role in outcome assessments or intervention development, and the study team had no ability to influence allocation assignments. A waiver of informed consent was obtained from both IRBs, and patients and providers are not directly consented for the study. Provider test ordering and patient completion of out-of-office BP testing will be analyzed according to the group to which their clinic had been assigned. To mask outcomes assessments, all effectiveness and implementation outcome assessments will be conducted by a multi-disciplinary team blinded to randomization assignment. Because of the nature of this trial providers and patients were not blinded.

#### Intervention and control conditions

Based on the theory-driven process described above, the final planned multi-faceted intervention for improving the completion of ABPM/HBPM consisted of (1) educational materials tailored to the clinic location, including one-time, in-person presentations to providers in a given clinic and about the USPSTF hypertension recommendations (e.g., discussion of hypertension screening in HIV patients, information on consequences of guidelines); (2) printed and online patient information materials on ABPM and HBPM; (3) information on how to order ABPM and HBPM for providers and staff via printed materials, website, and the one-time in-person presentation; (4) one-time in-person training/huddle for nurses on how to teach patients to conduct HBPM; (5) monthly to quarterly feedback via email/newsletters to providers about clinic-level success with appropriately ordering out-of-office testing for eligible patients and both clinic and provider-directed feedback on the outcomes of testing (e.g., ABPM completion and white coat hypertension detection rates); (6) an accessible, culturally adapted, and locally tailored ABPM clinical service to address access, reliability, and validity of ABPM; and (7) a CDS tool tailored to the local EHR (e.g., best practice advisory (BPA) in EPIC for Weill Cornell-affiliated clinics and an order set for referral to ABPM in Allscripts for Columbia-affiliated clinics) (Table 4). Outreach and educational activities (i.e., the implementation period) were conducted over a period of 6 months, and monthly to quarterly behavioral feedback, delivered via email, was designed to continue throughout the subsequent year. The CDS tools and ABPM testing service were designed to continue through the maintenance phase.

**Table 4:** Final components of intervention and usual care control arms

Multi-faceted implementation strategy intervention	Usual care control
• Educational presentations to primary care providers at grand rounds (one-time, in-person per clinic)	• Usual care
• Patient information materials on ABPM and HBPM (one-time printed material and website)	
• Training registered nurses to assist providers with teaching patients to conduct HBPM (one-time in-person training)	
• Information on how to order ABPM and HBPM to clinicians, nurses and front desk staff (one-time staff huddle and monthly–quarterly emails/newsletter)	
• A computerized EHR-embedded clinical decision support tool that prompts providers to recall the USPSTF hypertension guidelines and facilitates ordering of HBPM and ABPM for guideline-eligible patients (EHR change available throughout trial)	
• Monthly–quarterly feedback to primary care providers about clinic-level success with appropriately ordering ABPM and HBPM for eligible patients	
• An accessible, culturally adapted and locally tailored ABPM service (in-person ABPM service available throughout trial)	

*ABPM* ambulatory blood pressure monitoring, *HBPM* home blood pressure monitoring, *EHR* electronic health record

Clinics randomized to the usual care control condition continued to screen and diagnose hypertension according to their usual practice without the benefit of the EHR tools or other provider-directed intervention components. Patients from these clinics, however, were still eligible to receive ABPM from the locally available ABPM testing center if referred by their clinicians, though no special outreach regarding the availability of this service was made as part of this study.

#### Primary outcome

**Fig. 1:**
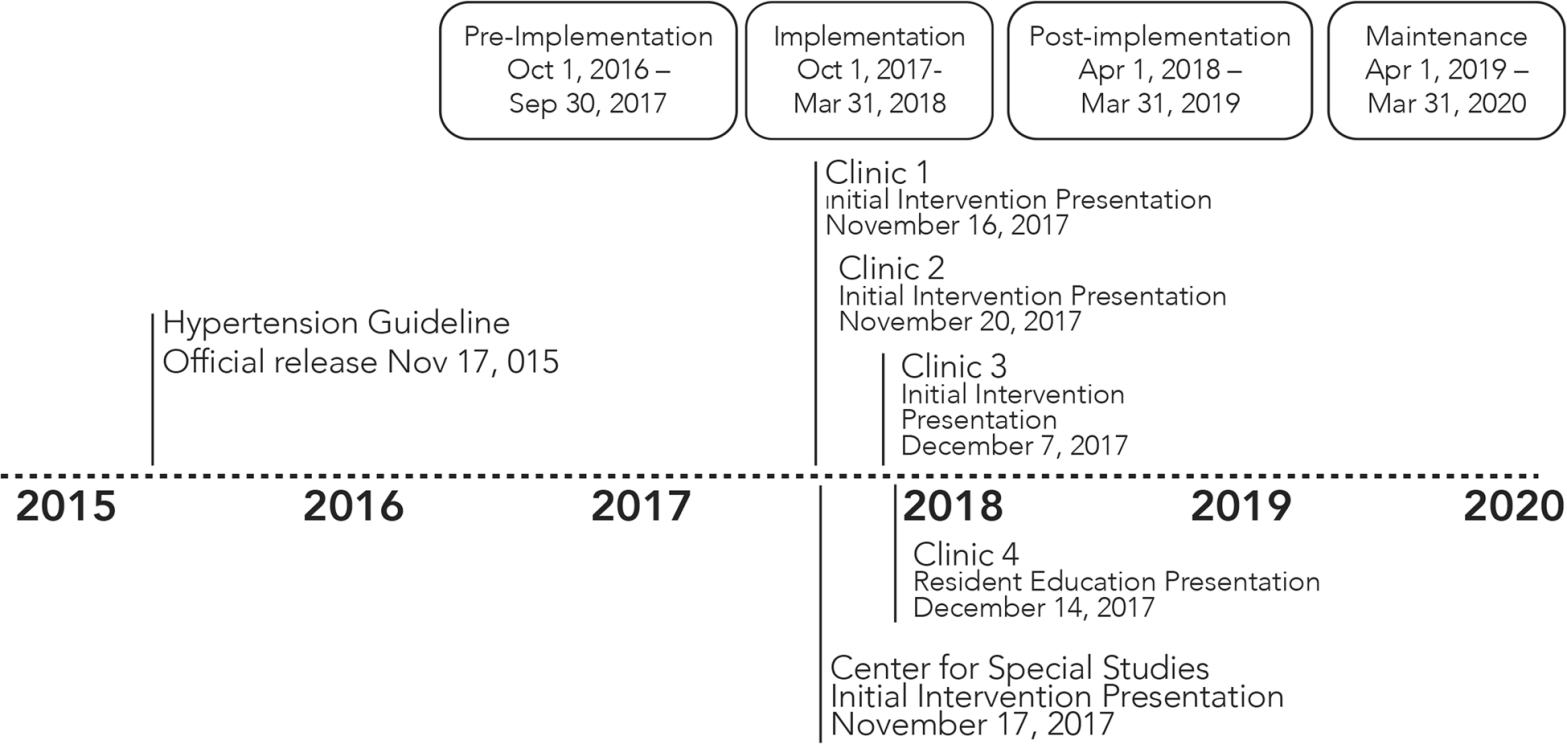
Study timeline

The primary outcome is the difference between intervention and control clinics in the change in the proportion of visits at which guideline-eligible patients complete out-of-office BP testing, either ABPM or HBPM, during the 12-month post-implementation period compared to the 12-month pre-implementation period (Fig. 1). For the primary analyses, the definition of what qualifies as an elevated office BP will be based on USPSTF recommendations (i.e., BP ≥ 140/90 mmHg) [34]. When multiple automated office BP readings are recorded during the same visit, the average of the BP readings will be used to classify office BP. Automated EHR queries will be used to identify potentially eligible patient-visits. Confirmation that visits meet eligibility criteria will be assessed through manual review of the EHR by medically trained abstractors blinded to group assignment using a standardized chart extraction form. Abstractors will review subsequent primary care visits for evidence of ABPM or HBPM test completion. ABPM testing will be coded as complete if sufficient awake BP readings (i.e., ≥ 10 awake BP readings) are available to estimate mean awake BP; asleep readings will not be required. Determination of ABPM test completion will be supplemented using data from the clinical ABPM testing service database. At least 10% of outcome assessments will be coded by a second assessor, with discrepancies resolved by consensus. If significant discrepancies are identified, the primary outcome assessor will undergo re-training, and prior assessments will be re-coded.

#### Secondary outcomes

Key secondary outcomes will include the difference between intervention and control clinics in the (1) pre-post change in the proportion of eligible patient-visits after which patients *complete* out-of-office BP testing during the *12-month maintenance period* (starts the last day of the post-implementation period); (2) pre-post change in the proportion of scheduled office visits where appropriate out-of-office BP testing is ordered, either ABPM or HBPM, during the 12-month post-implementation period and, separately, the 12-month maintenance period; and (3) pre-post change in the incidence of newly diagnosed white coat hypertension in the post-implementation and maintenance periods as determined by chart review. We will further test for variation in effectiveness by patient characteristics by conducting exploratory, stratified analyses that determine whether there are differences in the effect of the intervention according to patient age, sex, and HIV status.

#### Implementation and process outcomes

We will use the RE-AIM (Reach, Effectiveness, Adoption, Implementation, Maintenance) framework to evaluate our implementation strategy (Table 2) [35–38]. Reach will be ascertained by comparing the proportion and representativeness of eligible patients from intervention versus control clinics that complete out-of-office BP testing and by comparing characteristics (i.e., age, gender, ethnicity, HIV status) among eligible patients who do and do not complete out-of-office BP testing. Effectiveness will be determined by comparing the pre-post implementation change in the proportion of patients with elevated office BP who are referred for and complete out-of-office BP testing in intervention versus control group clinics (see “Primary outcomes” and “Secondary outcomes” sections above). Adoption will be based on the number of clinics approached who agree to randomization (all eight approached have already agreed) and by comparing the proportion and representativeness of providers from the intervention versus control group clinics who refer at least one patient for out-of-office BP testing. To ascertain maintenance, all primary and secondary effectiveness outcomes will be assessed during a 12-month maintenance period and pre-post changes in out-of-office BP testing will be compared between intervention and control clinics.

Implementation will be determined by the rosters of those who attend educational sessions; number of clinicians/clinic directors who send emails to their clinicians providing feedback on clinic-level out-of-office BP testing use and outcomes; proportion of nurses attending skills training on how to instruct patients in HBPM; number of times ABPM and HBPM materials are accessed online; and number of times the CDS tools (BPA at Weill Cornell-affiliated clinic; common order set at Columbia-affiliated clinic) are accessed.

To better understand the factors that facilitate or hinder success of implementation, we will also survey providers at the implementation and control clinics, 1 year after implementation. Specific goals will be to compare perceived attitudes and barriers toward the USPSTF hypertension screening recommendations among providers affiliated with intervention versus control clinics. Surveys will be informed by the same framework used in our formative work—the Theoretical Domains Framework [39] which explores constructs such as knowledge, self-efficacy, and perceived resources for out-of-office BP testing. Subsequent stakeholder interviews and/or focus groups will be conducted to further understand Proctor’s implementation outcomes (e.g., acceptability, feasibility, sustainability) as they relate to the intervention components [40] and to clarify barriers and facilitators to sustaining the intervention. These will be led by facilitators who are blinded to whether the clinic was assigned to the intervention or control group. Financial metrics (i.e., net revenue) will also be used to understand the sustainability of an ABPM testing service from an economic perspective.

#### Statistical approach

##### Effectiveness of implementation strategy

Three closely related hypotheses will be used to test the effectiveness:

1. The rate of out-of-office BP completion during the post-intervention period will be higher in clinics that received the intervention than in the control clinics.
2. The rate of out-of-office BP completion within clinics assigned to the intervention condition will be higher during the post-intervention period than during the pre-intervention period
3. The pre- to post-change in the likelihood of out-of-office BP testing will be greater in the clinics that received the intervention than in the control clinics.

A single multi-level Poisson regression model [41, 42] with three pre-specified contrasts, in which level 1 is the eligible patient and level 2 is the clinic will be used to test these hypotheses (see Additional file 2). The same approach will be used to evaluate the effect of the intervention on secondary outcomes including the rate of ABPM or HBPM referrals as well as to determine maintenance of the effect of the intervention in the second year after implementation (the maintenance period).

#### Process evaluation

Quantitative data will be analyzed using descriptive statistics to assess reach, adoption, and implementation outcomes relevant to the intervention group. Additionally, to assess adoption, multi-level Poisson regression models will be used to compare the proportion of clinicians that referred at least one patient for out-of-office BP testing in intervention clinics versus control clinics. To assess implementation, multi-level linear regression models will also be used to compare clinician ratings of perceived barriers and facilitators (7-point Likert scales) to ordering out-of-office BP testing for guideline-eligible patients from intervention clinics versus control clinics; as in the primary analyses, clinics will be treated as a random factor. The equivalent multi-level Poisson regression models will be used to compare intervention clinics versus control clinics in terms of clinician reports of whether each individual intervention components was received (yes/no). Finally, an intercept-only multi-level linear regression model will be used to estimate the average “helpfulness” of the intervention, rated on a 4-point Likert scale, for intervention clinics only. Content analysis will be used to evaluate transcripts of key stakeholder interviews or focus groups, if conducted (see Additional file 2).

### Power considerations

Power calculations are based on data from 2014; the year prior to the update to the USPSTF hypertension screening recommendations. Conservatively allowing that the rate of out-of-office BP completion in the usual care clinics increased in the years following the publication of the USPSTF hypertension screening recommendations to as high as 5% of patient visits with newly elevated office BP, we estimated the power to detect a 10% increase in out-of-office BP completion rate due to the intervention (i.e., relative risk 3.0; 15% vs 5%), at a two-tailed, α = 0.05 significance level, for each of the three hypotheses described above.

To estimate power, we simulated 10,000 datasets that each had the same number of (a) clinicians/clinic, (b) patients/clinician, and (c) visits/patient as the 2014 data. This was done for both the pre-intervention period and the post-intervention period. For each of the four pairs of matched clinics, one clinic was randomly assigned to the intervention condition and outcome data, incorporating the pre-specified intervention effect, were randomly generated. The multi-level Poisson regression analysis was performed on each of the 10,000 simulated datasets, and the proportion of datasets in which the null hypothesis was rejected, in the hypothesized direction, was an estimate of the statistical power to detect the pre-specified effect size. According to these simulations, the study has > 84% power to detect the hypothesized RR = 3.0 (15% completion rate in intervention clinics vs 5% in control clinics) for hypothesis 1, the comparison of post-intervention completion rates. The study also has > 92% power to detect the hypothesized RR = 3.0 (15% post-intervention completion rate vs 5% pre-intervention) for hypothesis 2, the test of the change in completion rate for intervention clinics only. Finally, the study has approximately 80% power to detect the hypothesized Condition*Period interaction effect (hypothesis 3) based on plausible assumptions about the variation in out-of-office BP testing completion rates between clinics and the within-condition correlation between pre-intervention and post-intervention clinic-level completion rates (see Additional file 2).

## Results

Medical directors of all eight eligible clinics agreed to participate in the study. All eight clinics were randomized, with four clinics assigned to the intervention group and four clinics to the control group (Table 5). The clinics are staffed by approximately 138 full- and part-time providers notable for diversity in provider training (including 16 general internists, 8 family medicine practitioners, 16 geriatricians, 27 HIV specialists, 11 NPs, and 60 GME trainees [44 housestaff, 16 fellows]). The clinics are also representative of diverse primary care patients, with some focused on providing care to people living with HIV and others focused on more general adult and older adult populations.

**Table 5:** Clinic characteristics^a^

Clinic characteristics	Clinic A	Clinic B
Pair 1		
Name	Farrell Community Health Center	Irving Sherwood Wright Center on Aging
Affiliate	Columbia	Cornell
Patient population	All ages	Older adults
Adult patient visits/year	21,000	9,800
Number of clinicians (training)	26 (8 family medicine, 18 housestaff)	21 (16 geriatrics, 1 NP, 4 fellows)
Trainees present	Yes	Yes
Pair 2		
Name	Rangel Community Health Center	Broadway Practice
Affiliate	Columbia	Columbia
Patient population	All ages	All ages
Adult patient visits/year	4800	7,000
Number of clinicians (training)	3 (3 internal medicine)	4 (3 internal medicine, 1 NP)
Trainees present	No	No
Pair 3		
Name	Washington Heights Family Health Center	Cornell Internal Medicine Associates Wright Center
Affiliate	Columbia	Cornell
Patient population	Adults	Adults
Adult patient visits/year	11,000	6000
Number of clinicians (training)	23 (7 internal medicine, 3 NP, 13 housestaff)	16 (3 internal medicine, 1 NP, 12 housestaff)
Trainees present	Yes	Yes
Pair 4		
Name	Comprehensive Health Program	Center for Special Studies
Affiliate	Columbia	Cornell
Patient population	People living with HIV	People living with HIV
Adult patient visits/year	7000	13,000
Number of clinicians (training)	32 (20 HIV, 7 fellows, 5 NPs)	13 (7 HIV, 5 fellows, 1 housestaff)
Trainees present	Yes	Yes

^a^To maintain blinding, clinic A and clinic B represent either intervention or control clinics

*HIV* human immunodeficiency virus, *NP* nurse practitioner

## Discussion

Although hypertension experts have long advocated for increased use of out-of-office BP testing, use of such testing in primary care as part of the diagnosis of hypertension remains infrequent [36, 43]. Inspired by the USPSTF guidelines, we employed a rigorous mixed-methods approach to develop a theory-informed multi-component implementation strategy with substantial potential for integrating out-of-office BP testing into routine primary care practice. All eight clinics we approached agreed to adopt the intervention if randomized (four have received the intervention) into our trial. These clinics serve a primarily lower-income, ethnically, and socioeconomically diverse population, including individuals living with HIV in whom hypertension and cardiovascular disease are the major cause of non-HIV-related morbidity and mortality. The results of our 1:1 cluster randomized trial will inform policy implementation efforts.

Recent efforts to develop an implementation strategy taxonomy have called for strategies informed by an understanding of the barriers to guideline uptake [44]. Using our formative work on barriers to out-of-office BP testing among providers in geographically diverse settings in the USAA [27], we applied the BCW framework, a theory-informed process for developing multi-component implementation strategies [45], to identify feasible intervention functions (e.g., training), policies (ABPM service), and corresponding behavioral change techniques (demonstration of behavior, adding objects to the environment) that would target barriers to uptake. We not only employed the BCW framework in intervention design but also utilized an innovative process of relying on a multi-disciplinary team of end users and experts in medicine, nursing, psychology, and qualitative methods to systematically determine the feasibility, applicability, and mode of delivery of each strategy component to arrive at what we believe is a more sustainable and generalizable strategy. Our preliminary results already suggest high adoption rates at the clinic level and also point the low acceptability of strategies, like intrusive CDS tools. Regardless, this protocol provides a roadmap for how best to rigorously map strategies to barriers and has the potential to inform the effectiveness of multi-level interventions that address both context and provider behavior.

The USPSTF, and more recently the ACC/AHA [43], recommendations for out-of-office BP testing have also highlighted the need for strategies that address context and resources integral to increasing the uptake of ABPM testing, the preferred out-of-office test according to these guidelines. These grade A USPSTF recommendations have informed Medicare and Medicaid screening test reimbursement policies, and reimbursement for ABPM was expanded to patients covered by Medicaid after the guideline update, a key barrier to prior ABPM testing in the past [46]. With guidelines and a reimbursement infrastructure has come the need for ABPM testing services (i.e., a service that supplies ABPM devices and synthesizes results for providers), particularly in low resource settings. Our study will improve the understanding of the impact of making ABPM testing more readily accessible to patients in resource-limited settings via a testing service. It will also provide an opportunity to assess sustainability, costs, reimbursement rates, and provider referral rates to an existing ABPM testing service within the current fiscal environment.

Given that ABPM testing is now reimbursed by some payers (e.g., Medicaid) [21], our study also supports the premise that provider-level barriers other than reimbursement underlie the underuse of ABPM for the diagnosis of hypertension [20], particularly provider concerns and assumptions related to cost-benefit ratios, patient discomfort, and validity [29, 47–49]. Our study provides an opportunity to assess the effect of addressing both context specific (e.g., ABPM availability) and provider level barriers (e.g., lack of knowledge, time, resources, concerns around valid or accurate testing, and beliefs about the consequences of testing) through CDS tools, patient and provider educational activities, and periodic provider feedback.

Furthermore, our findings will inform implementation of HBPM for the diagnosis of hypertension, which has become particularly salient in the post-COVID era of social distancing and telemedicine. While the USPSTF guidelines note that there is more evidence in support of ABPM as the best out-of-office BP testing method, they recommend ABPM or HBPM for confirming diagnoses of hypertension [9]. Our formative qualitative work identified overlapping yet distinct barriers to HBPM testing. Our trial will elucidate whether a primarily clinic and provider level intervention, which also includes supportive components around educating ancillary staff (e.g., nurses, medical assistances) on the proper protocol for HBPM and providing resources to facilitate the use of valid HBPM devices, will improve the uptake of HPBM for hypertension diagnosis. Our mixed-methods approach to assessing outcomes will identify differences in HBPM and ABPM implementation processes essential for guiding policy.

If proven effective, our results could inform the ways in which policy-makers, implementation scientists, and primary care clinics seek to increase the uptake of out-of-office BP testing guidelines not just for hypertension diagnosis but for the management of hypertension as well. Our results will also broadly contribute to the understanding of solutions needed to address gaps in the implementation of other key screening guidelines. Out-of-office BP testing represents a paradigm shift in hypertension diagnosis, and regardless of the outcome of this trial, our study will generate critical information on the feasibility, resources, and implementation processes needed to implement these guidelines, particularly in the post COVID-era. We will further elucidate implementation processes, such as costs, reimbursement, referrals to, and use of an ABPM service while also identifying strategies effective in improving provider use of out-of-office testing in resource limited settings. Finally, our results will have direct applicability to the use of testing among patients from low-income and racial and ethnic minority groups who have historically low uptake of evidence-based guidelines (perhaps due to provider and patient factors) [50] as well as high rates of both sustained and white coat hypertension [51].

### Limitations

Our study has enrolled clinics primarily serving diverse, economically vulnerable populations in New York City, and this may limit generalizability to clinics serving other patient populations. Our clinics use two different EHRs, and the CDS tools we developed may not be easily transferable to clinics using other electronic systems. We will rely on EHR data for our primary outcomes which may be affected by missing data or misclassification, though we have created a process for masking outcomes assessments and monitoring for high-quality coding. EHR data are also increasingly used in implementation science trials and allow for a pragmatic approach to assessing effectiveness compared with enrolling individual patients and providers. While our intervention primarily targets provider test ordering, the effectiveness of our intervention on ABPM and HBPM completion rates may be more contingent on patient factors which are only indirectly targeted. Nonetheless, our intervention targets several “opportunity” barriers, such as access to an ABPM testing service, that contribute to provider behavior, which we hypothesize is crucial to completion rates.

## Conclusions

An important contribution of this project has been the identification of theory-informed implementation strategy components needed to optimize the uptake of HBPM and ABPM as part of hypertension screening in an ethnically, racially, and socioeconomically diverse patient population. We have identified a promising, disseminatable multi-component implementation strategy, which includes provider education, reminders, clinic-level feedback to clinicians on testing use and outcomes, clinical decision support (CDS) tools, nursing training on HBPM, and access to ABPM testing services. The impact of these strategies will be formally tested in the on-going EMBRACE cluster randomized trial across eight primary care clinics.

## Supplementary information

**Additional file 1.** Behavior Change Wheel: Multistep process for intervention development.

**Additional File 2.** Detailed statistical analysis plan and power considerationsstatistical approach.

## Data Availability

The datasets used and/or analyzed during the current study are available from the corresponding author on reasonable request.

## Abbreviations

ABPM: Ambulatory Blood Pressure Monitoring
AACN: Ambulatory Care Network which is a large primary care network which is the setting for the intervention
BP: Blood pressure
CDS: Clinical Decision support
EHR: Electronic Health Record
HBPM: Home blood pressure monitoring
NYPH: New York-Presbyterian Hospital
PCP: Primary Care physician the primary medical provider for a patient in the primary care setting
RCT: Randomized controlled trial
USPSTF: US Preventive Services Task Force
