# Supplementary material for: Design and study protocol for a cluster randomized trial of a multi-faceted implementation strategy to increase the uptake of the USPSTF hypertension screening recommendations: the EMBRACE study
Source: Implement Sci. 2020 Aug 8;15:63. doi: 10.1186/s13012-020-01017-8 (PMC7414682; doi:10.1186/s13012-020-01017-8)

SUPPLEMENTARY MATERIAL

Additional File 1. Behavior Change Wheel: Multistep process for intervention development


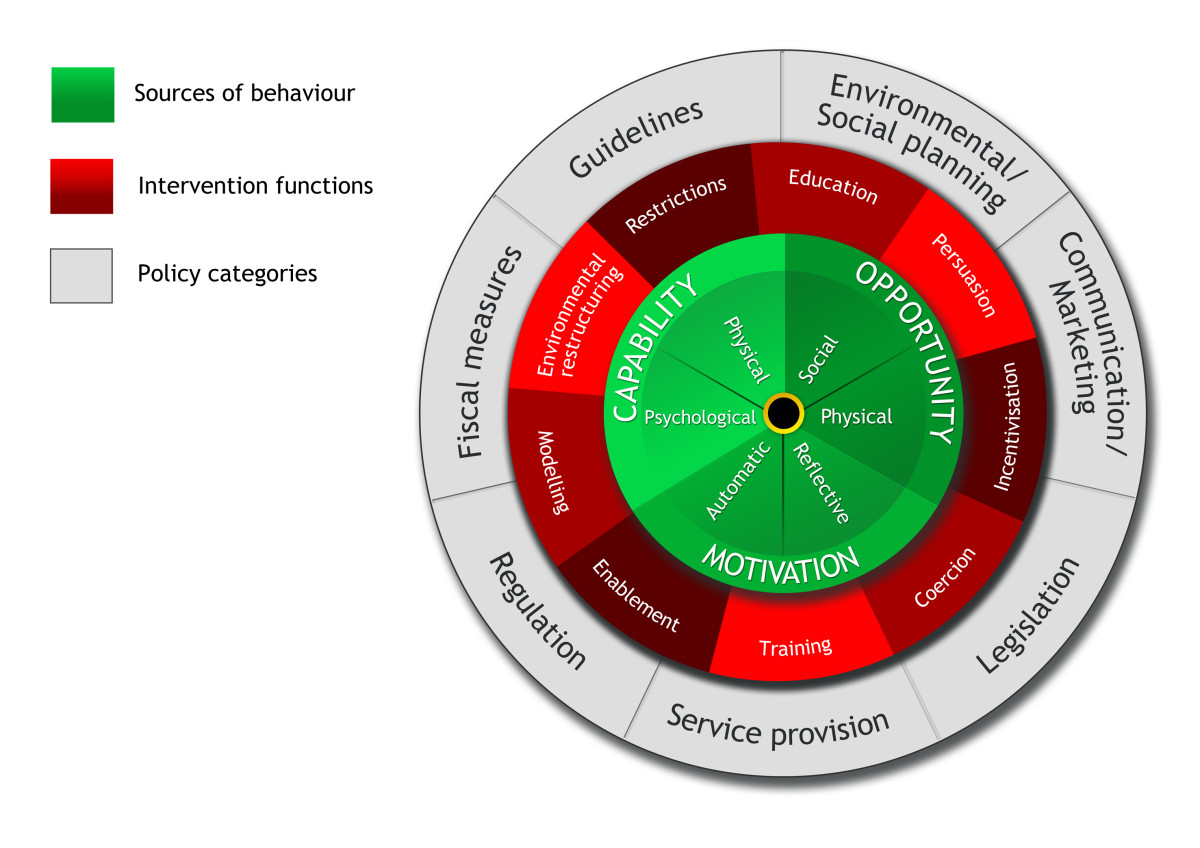

Supplement: Supplementary file 1 — Additional file 1. Behavior Change Wheel: Multistep process for intervention development. [file 13012_2020_1017_MOESM1_ESM.docx]
