# Supplementary material for: Design and study protocol for a cluster randomized trial of a multi-faceted implementation strategy to increase the uptake of the USPSTF hypertension screening recommendations: the EMBRACE study
Source: Implement Sci. 2020 Aug 8;15:63. doi: 10.1186/s13012-020-01017-8 (PMC7414682; doi:10.1186/s13012-020-01017-8)
Supplement: Supplementary file 2 — Additional File 2. Detailed statistical analysis plan and power considerationsstatistical approach. [file 13012_2020_1017_MOESM2_ESM.docx]

**Additional File 2.** **Detailed Statistical Analysis Plan and Power Considerations**

**Statistical Approach.**

*Effectiveness of intervention.* The relative change, from the 12-month pre-intervention period to the 12-month post-intervention period, in the proportion of patient visits after which guideline-eligible patients complete out-of-office BP testing (either ABPM or HBPM) will be the primary outcome measured at the clinic level. This change will be estimated using a multilevel Poisson regression model(1, 2) in which level 1 is the eligible patient and level 2 is the clinic. There will be two observations for each of the 8 clinics, one for the pre-intervention and one for the post-intervention 12-month period, and each observation will include:

1. the outcome: the number of patients who completed out-of-office BP testing within six months of an eligible visit that occurred within the given 12-month period,
2. the total number of unique eligible patient-visits during the given 12-month period (used as an “offset” variable, after log-transformation, in the Poisson regression), and
3. two binary predictors, Condition (0=control site, 1=intervention site) and Period (0=pre-intervention, 1=post-intervention).

The model will include two correlated random intercepts, one to capture site-specific differences (i.e., clustering by site) during the pre-intervention period and one for the post-intervention period. We considered a much more complex model that would include random effects for each individual physician, but recent work suggests that it is sufficient to model only clustering at the highest level of analysis at which clustering is assumed to occur.(3) Using a log link function, we will estimate the following model:

ln(OOO_BP*_sp_*) = α*_sp_* + β_1_*Condition*_s_* + β_2_*Period*_p_* + β_3_*Condition*_s_**Period*_p_* (1)

Where, *s* indexes clinic sites, *s* = 1 to 8,

*p* equals 0 for Pre-intervention period, 1 for Post-intervention period,

OOO_BP*_sp_* equals the number of out-of-office BP assessments at site s during period p,

α_s1_ equals the site specific effect for the pre-intervention period,

α_s2_ equals the site specific effect for the post-intervention period, and

α_s1_ and α_s2_ are assumed to have equal standard deviations, σ, and a bivariate normal distribution with correlation *r*.

This analysis will be used to test three closely related hypotheses:

1. The rate of out-of-office BP completion during the post-intervention period will be higher in clinics that received the intervention than in the control clinics. Exp(β_1_ + β_3_) equals the relative risk (RR) of out-of-office BP assessment in intervention compared to control clinics during the post-intervention period, and testing the hypothesis that β_1_ + β_3_ = 0 is equivalent to testing the hypothesis that this RR=1.0.
2. The rate of out-of-office BP completion within clinics assigned to the intervention condition will be higher during the post-intervention period than during the pre-intervention period. Exp(β_2_ + β_3_) equals the RR of out-of-office BP assessment in during the post-intervention period compared to the pre-intervention period, within the clinics assigned to the intervention condition, and testing the hypothesis that β_2_ + β_3_ = 0 is equivalent to testing the hypothesis that this RR=1.0.
3. The Pre- to Post- change in the likelihood of out-of-office BP testing will be greater in the clinics that received the intervention than in the control clinics. β_3_, the coefficient of the interaction term, estimates the extent to which the Pre- to Post- change in the likelihood of out-of-office BP testing is greater in the clinics that received the intervention than in the control clinics. More specifically, if RR_0_ equals the relative risk of out-of-office BP completion in intervention clinics compared to control clinics during the pre-intervention period and RR_1_ equals the relative risk during the post-intervention period, then exp(β_3_) equals RR_1_/RR_0_, and testing the hypothesis that exp(β_3_) =0 is equivalent to testing the hypothesis that RR_1_/RR_0_ = 1.

Although we anticipate that the matching of clinics will have promoted balance between those clinics assigned to the intervention and control conditions, we will not incorporate the matching into the analysis. The same approach will be used to evaluate the effect of the intervention on secondary outcomes including the rate of ABPM or HBPM referrals as well as to determine maintenance of the effect of the intervention in the second year after implementation (the maintenance period).

*Power Considerations*

Our power calculations are based on data from 2014, the year prior to the update to the USPSTF hypertension screening recommendations. That year, there were 770 unique patients, with 997 eligible patient visits, seen by 169 clinicians at the 8 participating clinics. Our power calculations assume this same 2014 distribution of clinicians, patients, and eligible visits/patient for both pre- and post-intervention 12-month monitoring periods. For the effect size, we estimate that even with the release of the USPSTF guideline, there continued to be very low uptake of out-of-office BP testing to confirm hypertension diagnoses in wait-list clinics. Conservatively allowing that the rate of ABPM completion in the wait-list clinics increased in the years following the publication of the USPSTF hypertension screening recommendations to as high as 5% of patient visits with incident hypertension, we estimated the power to detect a 10% increase in out-of-office BP completion rate due to the intervention (i.e., 15% vs 5%), at a two-tailed, α=0.05 significance level, for each of the 3 hypotheses described above. In addition to the hypothesized out-of-office BP completion rates (5% and 15%), the power is influenced by two parameters associated with the random effects (i.e., clustering by clinic): 1) the standard deviation, σ, of the systematic differences variability among clinics in their log-transformed rates of out-of-office BP completion rates [σ = ln(CV), where CV, coefficient of variation is the standard deviation across clinics of their out-of-office BP completion rates divided by the overall average completion rate; e.g., if CV=0.40, then the clinic-specific completion rates have a standard deviation of 2% when the overall average pre-intervention completion rate equals 5% and a standard deviation of 6% when the overall average post-intervention completion rate for clinics assigned to the intervention condition equals 15%], and 2) the correlation, *r*. When CV=0, there are no systematic differences among clinics (i.e., no clustering by clinic) and the model becomes a fixed effects, rather than a random effects, model. As CV increases, the systematic differences among the clinics increases, reducing the ability (i.e., power) to generalize beyond the 8 participating clinics. On the other hand, increasing levels of *r* increase the power to detect pre-intervention to post-intervention change, because it reduces the standard deviation of α_s2_ - α_s1_. We varied

For CV=0 and each combination of CV=0.10, 0.20, 0.30 and 0.40 with *r*=0.50, 0.60, 0.70, 0.80, and 0.90, we ran two sets of 10,000 simulations. For each simulation,

1. one clinic (within each pair of matched clinics; see Table 5) was randomly assigned to the intervention and the other to the control condition,
2. α_s1_ and α_s2_ were randomly generated from a log-bivariate normal distribution with the specified CV and *r* (thereby introducing the targeted amount of systematic variability among clinics), and then,
3. using the 2014 data for each individual clinic, each eligible 2014 patient-visit within that clinic was randomly assigned a value of 1 (having out-of-office BP) if a randomly generated value from a uniform(0,1) distribution was <exp(α_s1_) for pre-intervention period or <exp(α_s2_ + β_3_ * Condition) for post-intervention period (β_1_ and β_2_ were both assumed to equal zero); those visits not assigned a value of 1 were assigned a value of 0. Each 2014 patient-visit was used to generate a random observation for that clinic for the pre-intervention period and a random observation for the post-intervention clinic. Within a period, the generation of outcomes was constrained such that once a patient completed out-of-office BP, any subsequent eligible clinic visits for that patient were ineligible for randomization.

The multilevel Poisson regression analysis was performed on each of the 10,000 simulated datasets, and the proportion of datasets in which the null hypothesis was rejected, in the hypothesize direction, was an estimate of the statistical power to detect the assumed effect size. To repeat, 2 sets of 10,000 simulations were performed for each combination of CV and *r*. The results are shown in Figure 2. In each panel, the solid red horizontal line shows the power if CV=0. Panel A shows that for all considered values of CV and *r*, the study has >84% power to detect the hypothesized RR=3.0 (15% completion rate in intervention clinics vs 5% in control clinics; power >92% if CV≤0.30) for hypothesis 1, the comparison of post-intervention completion rates. Panel B shows that for all considered values of CV and *r*, the study has >92% power to detect the hypothesized RR=3.0 (15% post-intervention completion rate vs 5% pre-intervention) for hypothesis 2, the test of the change in completion rate for intervention clinics only. Finally, Panel C shows that for CV=0, the study has 84% power to detect the hypothesized Condition*Period interaction effect (hypothesis 2), that the power drops to slightly less than 80% for low values of CV and high values of *r*, and decreases to as low as 65% for CV=0.40 and *r*=0.50.

*Accounting for Clustering:* Overall, we incorporated between-site variability in out-of-office BP completion rates at both baseline and follow-up (with variability equal to 0, 10%, 20%, 30% or 40% of the average rate), and a correlation between the site-specific rates of r=0.50, 0.60, 0.70, 0.80, or 0.90.  The fact that r was assumed to be <1.00 means that unlike most power analyses, we not only incorporated between-site variability in average levels, but we also incorporated between-site variability in change from baseline to follow-up. Thus, GEE cannot be used to perform the analysis because there are two random effects which can be viewed, equivalently, either as Site-specific differences (clustering) at baseline and Site-specific differences at follow-up, OR as Site-specific differences at baseline and Site-specific differences in change from baseline to follow-up.

**Figure 2. Power estimates for three hypothesized tests of the intervention’s effect on out-of-office blood pressure completion rates.**

**
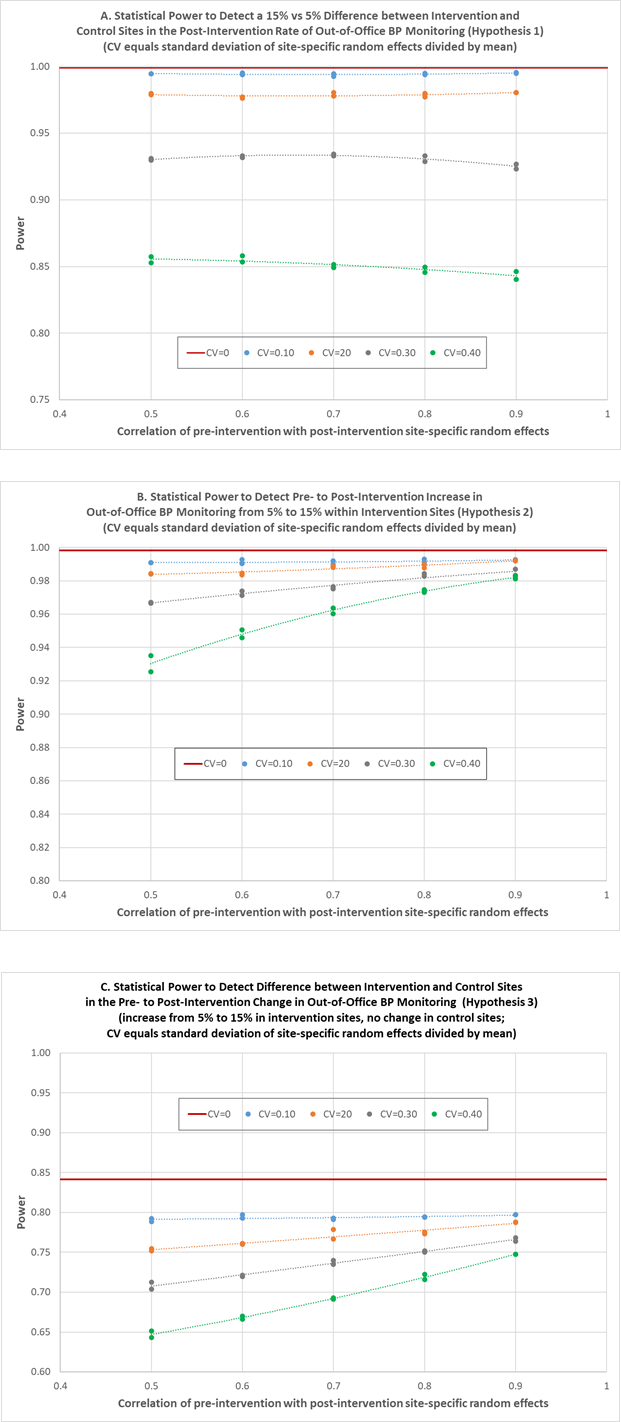
**

**References**

1. Singer JD, Willett JB. It’s about time: Using discrete-time survival analysis to study duration and the

timing of events. *J Educ Stat*. 1993;18:155-195.

2. Hedeker D, Siddiqui O, Hu FB. Random-effects regression analysis of correlated grouped-time survival data. *Stat Methods Med Res*. 2000;9:161-179.

3. Murray DM, Hannan PJ, Baker WL. A Monte Carlo study of alternative responses to intraclass correlation in community trials. Is it ever possible to avoid Cornfield's penalties? *Eval Rev*. 1996;20:313-337.
